# *oqxAB*-Positive IncHI2 Plasmid pHXY0908 Increase *Salmonella enterica* Serotype Typhimurium Strains Tolerance to Ciprofloxacin

**DOI:** 10.3389/fcimb.2019.00242

**Published:** 2019-07-03

**Authors:** Xinlei Lian, Xiran Wang, Xiao Liu, Jing Xia, Liangxing Fang, Jian Sun, Xiaoping Liao, Yahong Liu

**Affiliations:** ^1^National Risk Assessment Laboratory for Antimicrobial Resistance of Animal Original Bacteria, South China Agricultural University, Guangzhou, China; ^2^Laboratory of Veterinary Pharmacology, College of Veterinary Medicine, South China Agricultural University, Guangzhou, China; ^3^Guangdong Provincial Key Laboratory of Veterinary Pharmaceutics Development and Safety Evaluation, South China Agricultural University, Guangzhou, China

**Keywords:** *oqxAB*, IncHI2, plasmids, *Salmonella*, tolerance

## Abstract

*Salmonella enterica* serotype Typhimurium is a major global food-borne pathogen and causes life-threatening infections. Although the resistance mechanisms to fluoroquinolones in *S*. Typhimurium had been well-defined, tolerance to fluoroquinolones and the associated mechanism for this are obscure. In the current work, we investigated an *oqxAB*-positive plasmid pHXY0908 and analyzed its role in *S*. Typhimurium tolerance to ciprofloxacin using time-kill, transcriptome sequencing and real-time PCR. *S*. Typhimurium ATCC14028 could survive under lethal concentrations of ciprofloxacin after acquiring plasmid pHXY0908. Transcriptome sequence analysis showed the chromosomal genes were systematically regulated after acquiring this plasmid suggesting an interaction between chromosome and plasmid. Additionally, the chromosomal efflux pump genes *acrB, acrA, tolC*, and *yceE* were up-regulated after acquiring plasmid pHXY0908 suggesting that these efflux pumps may contribute to the survival of ATCC14028 exposed to the lethal concentrations of ciprofloxacin. In conclusion, this is the first known report demonstrating that an IncHI2 type plasmid harboring *oqxAB* could assist *S*. Typhimurium survival under lethal concentrations of ciprofloxacin.

## Introduction

*Salmonella enterica* Typhimurium is a major global food-borne pathogen, causing a wide spectrum of human and animal diseases including acute gastroenteritis, bacteremia, and extra intestinally localized infections involving many organs (Coburn et al., 2007). Poultry, pigs, cattle, and reptiles are *S*. Typhimurium reservoirs and humans generally become infected by eating undercooked or contaminated food (Gomez et al., 1997).

Although intestinal infections caused by non-typhoid *Salmonella* are usually self-limiting, effective antimicrobial therapy is essential if invasive infection occurs (Hohmann, 2011). The extensive use of antimicrobials in humans and animals has led to an increase in multi-drug resistance among numerous bacterial strains. In particular, multidrug resistant (MDR) *Salmonella* isolates such as *S*. Typhimurium monophasic variant (*S*. 4,[5],12:i:-), *S*. Typhimurium DT104 and *S*. Rissen are major global public health problems (Threlfall, 2000; Hopkins et al., 2010; Mather et al., 2013; Gomes-Neves et al., 2014). Due to increasing resistance to the conventional antimicrobial agents such as ampicillin, chloramphenicol, and trimethoprim/sulfonamides, fluoroquinolones such as ciprofloxacin for the treatment of severe invasive salmonellosis has become more common (Hohmann, 2011). The use of fluoroquinolones has also led to a rapid increase in reduced susceptibility of *S*. Typhimurium to these therapeutics. MDR *S*. Typhimurium with reduced ciprofloxacin susceptibility has become common in China (Li et al., 2013a; Wong and Chen, 2013).

Resistance to fluoroquinolones is mainly due to point mutations in the quinolone resistance-determining region (QRDR) of the gyrase (*gyrA* and *gyrB*) and topoisomerase IV (*parC* and *parE*) genes. The efflux pump AcrAB-TolC can decrease susceptibility to drugs from seven antibiotic classes such as fluoroquinolones, tetracyclines, rifamycins, oxazolidinones, macrolides, and so on (Schuster et al., 2017). The efflux function of AcrAB-TolC is not only used for antibiotics but also small molecules, such as metabolins, dyes, detergents, organic solutes and even bile salts (Nikaido, 1996; Pos, 2009; Oswald et al., 2016). In addition, plasmid-mediated quinolone resistance (PMQR), including derivatives of quinolone resistance proteins (Qnr), aminoglycoside acetyltransferase Aac(6′)-Ib-cr, and quinolone efflux pumps QepA and OqxAB, have also been described in fluoroquinolone-resistant *S*. Typhimurium isolates (Strahilevitz et al., 2009; Poirel et al., 2012). Additionally, tolerance to ciprofloxacin has been observed that was primarily linked to mutations in *gyrA* and *parC* (Dahiya et al., 2014). Even though tolerance of *Salmonella* is most often attributed to the action of efflux pumps the evidence linking PMQR genes and fluoroquinolone tolerance is scant (Webber et al., 2006; Thorrold et al., 2007).

Plasmids play an important role in the dissemination of antimicrobial resistance genes (Carattoli, 2009) and different incompatibility group plasmids have been examined for their roles in MDR *S*. Typhimurium (Fernandez et al., 2007; Zaidi et al., 2011). In our previous study, we identified that spread of *oqxAB* was predominately due to transferable MDR IncHI2 pHXY0908-like plasmids in *S*. Typhimurium (Li et al., 2013a). Interestingly, we found that possession of plasmid pHXY0908 was correlated with the treatment failure to avian salmonellosis using enrofloxacin at a routine dosage. This was in spite of the fact that this plasmid could directly confer only low-level fluoroquinolone resistance (Chen et al., 2016). In the current study, we examined potential mechanisms involved in *S*. Typhimurium survival to the ciprofloxacin selective pressure after acquisition of plasmid pHXY0908.

## Materials and Methods

### Bacterial Strains

*S*. Typhimurium ATCC14028 and *S*. Typhimurium ATCC14028-bearing plasmid pHXY0908 (ATCC14028-pHXY0908) were used as the test strains in the present study. IncHI2 plasmid pHXY0908 harboring *oqxAB* confers a multi-drug resistance phenotype. The ATCC14028-bearing plasmid pHXY0908 were obtained by electroporation of the transferable pHXY0908 into *S*. Typhimurium ATCC14028 as previously described (Chen et al., 2016, 2017).

### Minimum Inhibitory Concentration (MIC) and Mutant Prevention Concentration (MPC) Determinations

The MIC of ciprofloxacin was determined for *S*. Typhimurium ATCC14028 and *S*. Typhimurium ATCC14028-bearing plasmid pHXY0908 by the standard broth microdilution methods according to the recommendations of the Clinical and Laboratory Standards Institute (CLSI) (M100-S25). The breakpoint criteria used to determine ciprofloxacin phenotype in *Salmonella spp*. was based on the CLSI breakpoint criteria [ ≤ 0.06 μg/mL (susceptible), 0.12–0.5 μg/mL (intermediate), and ≥1 μg/mL (resistant)]. *E. coli* ATCC25922 was used as a quality control strain.

The MPC values were determined as previously described (Allou et al., 2009). In summary, each strain was grown at 37°C in antibiotic-free Mueller Hinton (MH) broth for ~6 h, until an OD_600_ of ~1.0 was reached (corresponding to ~10^9^ CFU/mL). Cultures (20 mL) were centrifuged at 4,000 × g for 15 min. The supernatant was discarded and the pellet containing ~10^10^ CFU/mL was suspended in 2 mL of sterile MH broth. MH agar plates containing ciprofloxacin at levels ranging from 0.002 to 32 μg/mL (diluted in log_2_ series) against each strain were inoculated with 100 μL of cell suspension and incubated at 37°C for 96 h. The MPC was recorded as the lowest ciprofloxacin concentration at which no colonies grew on an agar plate after 96 h.

### Time-Kill Experiments

Time-kill curve kinetics assays were conducted using MH broth containing ciprofloxacin levels equaling 1 × MIC, 2 × MIC, 4 × MIC, and 8 × MIC of the strains tested. Antibiotic-free broth was evaluated in parallel as a control. Cultures were incubated at 37°C with shaking. Viable counts were determined by serial dilution after 0, 3, 6, 9, and 24 h of incubation and by plating 100 μL of the control, test cultures, or with dilutions at the indicated times onto MH agar plates. Colony counts were determined after 24 h of incubation.

### Plasmid Sequencing

DNA of plasmid pHXY0908 was sequenced using the Single Molecule Real Time (SMRT) DNA Sequencing approach. After filtering *S*. Typhimurium ATCC14028 chromosomal DNA data, the remaining reads were assembled by HGAP2.2.0 method (Chin et al., 2013). Open reading frames (ORF) were predicted using ORF Finder (http://www.ncbi.nlm.nih.gov/gorf/gorf.html) and annotation was performed using RAST tools (Aziz et al., 2008). The sequence comparison and map generation was performed using BLAST (http://blast.ncbi.nlm.nih.gov) and Easyfig version 2.1 (Sullivan et al., 2011). The annotated sequence of pHXY0908 has been submitted to the GenBank nucleotide sequence database under the accession number KM877269.

### Transcriptome Sequencing and Sequence Analysis

ATCC14028 and ATCC14028-pHXY0908 were cultured in LB broth with and without 1/2 × MIC levels of ciprofloxacin. Total RNA was extracted as previously described (Li et al., 2013b). A pooled sample from 3 independent experiments was used for RNA-seq. Ribo-Zero rRNA Removal Kit (Gram-Negative Bacteria) (Epicentre, Madison, WI, USA) was used to remove rRNA from total bacterial RNA. The library was constructed using an Illumina TruSeq RNA sample Prep Kit v2 as previously described (Wang et al., 2013). In brief, mRNA was fragmented into lengths of 200~300 bp and first and second strand cDNA was synthesized. The short cDNA fragments were purified and end repaired and tailed with single A (adenine) addition. Adapters were ligated to the A-tailed cDNA fragments and ligated. These cDNA fragments were enriched by 12 PCR cycles. Purified libraries were quantified using a by Qubit 2.0 Fluorometer (Invitrogen, Carlsbad, CA, USA) and validated using an Agilent 2100 Bioanalyzer (Agilent, Beijing, China). Libraries were sequence by the Illumina Hiseq-2000 for 90 cycles. The reads that passed the Illumina quality filter were kept for sequence analysis.

High quality reads were mapped to *S*. Typhimurium strain 14028 genome (downloaded from https://www.ncbi.nlm.nih.gov/nuccore/267991652/) and plasmid pHXY09080 sequence by using SOAP aligner/SOAP2 (Li et al., 2009) with 5 max alignment error. The mRNA abundance was normalized using rpkM (reads per kilobase per million reads) (Mortazavi et al., 2008). Gene differential expression analysis was performed as previously described (Audic and Claverie, 1997). The genes with <0.001 FDR and >2-fold change or <0.5-fold were detected as differentially expressed genes (DEG). The RNA-seq data had been submitted to SRA database (Accession number: PRJNA544622, https://www.ncbi.nlm.nih.gov/Traces/study/?acc=PRJNA544622).

### Gene Ontology Enrichment Analysis, Kyoto Encyclopedia of Genes and Genomes (KEGG) Pathway Enrichment Analysis, and Functional Protein Association Networks Analysis

The DEGs were performed Gene Ontology enrichment analysis by MATLAB bioinformatics toolbox (MathWorks, Natick, MA, USA). The gene ontology annotations were downloaded from Gene Ontology Consortium (http://www.geneontology.org/page/download-go-annotations). KEGG pathway enrichment analysis was performed by ClueGO and CluePedia which are Cytoscape apps (Bindea et al., 2009, 2013). The functional protein association networks were constructed by STRING with high confidence and hided disconnected nodes in the network (Szklarczyk et al., 2017).

### Validation the DEGs by qRT-PCR

Total bacterial RNA was obtained and reverse transcribed into cDNA as described (Li et al., 2013b). The qRT-PCR was performed with SYBR Premix Ex Taq (Takara, Dalian, China) in an iQ5 thermal cycler (Biorad, Hercules, CA) according to the manufacturer's instructions. The cycling conditions were as follows: 94°C for 5 min, followed by 35 cycles at 94°C for 1 min, at 55°C for 1 min and 72°C for 1 min with a final step of 72°C for 5 min. Melting curves were read from 60 to 95°C in steps of 1°C. Normalized expression levels of the target gene transcripts were calculated relative to 16S rRNA using the –ΔΔCT method. The primers used for gene amplification are listed in Table S1.

### Statistics

All the *in vitro* experiments described above were repeated at least three times. Geometric means were used to express the results for MICs and MPCs and the means ± standard deviations (SD) were calculated for CFU counts.

## Results

### MIC, MPC, and Time-Kill Curve Assays

Ciprofloxacin MIC and MPC values were increased by 4-fold when *S*. Typhimurium ATCC14028 acquired plasmid pHXY0908. However, the MPC/MIC ratios between these strains did not differ (Table 1).

**Table 1:** Ciprofloxacin MICs and MPCs of the *Salmonella* Typhimurium strains used in this study.

**Strain**	**MIC (mg/L)**	**MPC (mg/L)**	**MPC/MIC ratio**
ATCC14028	0.03	0.5	16
ATCC14028-pHXY0908	0.125	2	16

*Each value is the mean ± SD derived from three independent experiments*.

**Figure 1:**
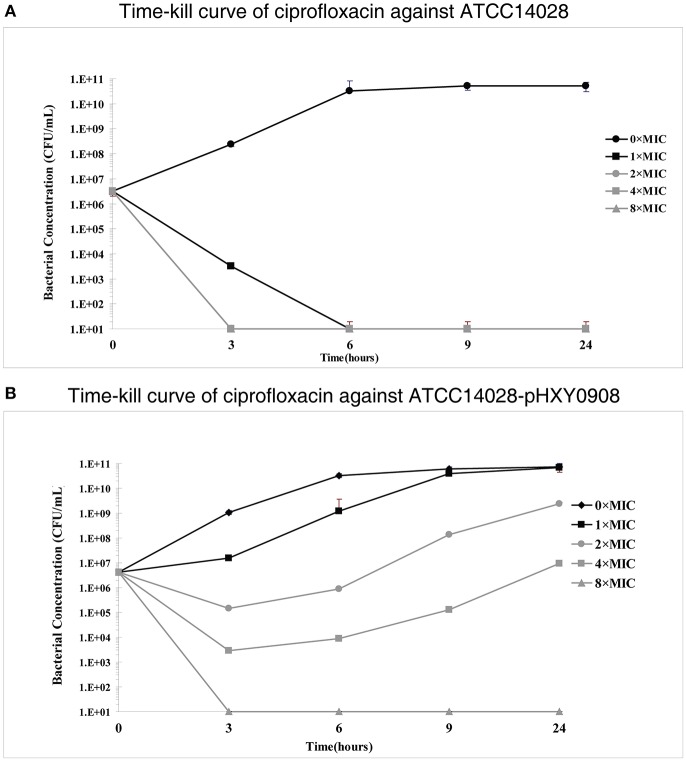
*In vitro* time-kill studies of ciprofloxacin **(A)** *Salmonella* Typhimurium ATCC14028 and **(B)** and ATCC14028-pHXY0908 1 × MIC is equivalent to 0.03125 mg/L for ATCC14028 and 0.125 mg/L for ATCC14028-pHXY0908. The lines and markers of 2 × MIC and 8 × MIC coincided with 4 × MIC in **(A)**.

However, time-kill curve assays indicated a survival advantages at 1×, 2×, and 4× ciprofloxacin MIC levels for the plasmid bearing strain compared to the parental strain. In addition, the antibacterial activity of ciprofloxacin significantly decreased over the 3 h antibiotic exposure period against ATCC14028 strain harboring plasmid pHXY0908. Interestingly, the bacterial concentration of ATCC14028-pHXY0908 increased after 3 h and after 6 h under the 1 × MIC. The CFU of ATCC14028-pHXY0908 was almost identical with ATCC14028-pHXY0908 cultured without ciprofloxacin for 24 h (Figure 1).

### General Features of Plasmid pHXY0908

**Figure 2:**
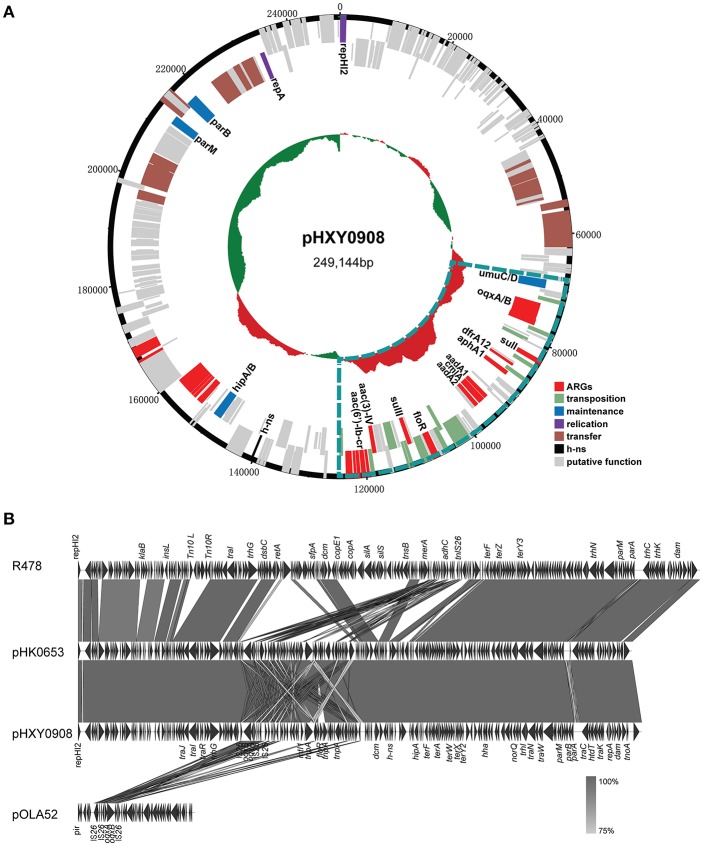
Genetic map and linear comparisons for pHXY0908. **(A)** Genetic map of pHXY0908. The central region of the map is G+C plot of the nucleotide sequence. Boxes represent ORFs predicted with RAST tools and are color coded after their predicted functions. The blue lines represent the multidrug-resistant regions. **(B)** Linear comparison of pHXY0908, R478, pHK0653, and *oqxAB*-positive plasmid pOLA52. The arrows represent ORF position and transcriptional direction. Regions of homology are shaded in gray and functional regions are indicated above and below the linear maps.

Plasmid pHXY0908 is a circular molecule of 249,144 bp with an average G + C content of 46.22% and harboring 265 predicted ORFs (Figure 2A). The backbone region of pHXY0908 is closely related to other IncHI2 plasmids including pHK0653 (KT334335) from *Salmonella* enterica, plasmid R478 (BX664015) from *Serratia marcescens* and pAPEC-O1-R (DQ517526) from an extraintestinal pathogenic *Escherichia coli* (Figure 2B). These backbone regions contained the replication gene *repHI2*, the plasmid maintenance and partitioning modules *parA-parB* and *hipAB* and two *tra* transfer regions. In addition to the typical backbone, the 52,876 bp MDR region of pHXY0908 contained 9 IS*26* elements flanking four main segments (Figure S1). The first segment containing an *oqxAB* cassette flanked by IS*26* elements was first identified in the IncX1 type plasmid pOLA52 from *E. coli*. The second segment between IS*26*-6 and IS*26*-7 contained the antibiotic resistance genes *sul3, aadA1, cmlA*, and *aadA2* and was identical to plasmid pND11_107 (HQ114281). The third segment containing insertion sequences IS*26*-7 and IS*26*-8 and the structure *tnp21*-*floR*-*sul2*-IS4-*aacC4*-IS*26*, identical to that of pK1HV (HF545434) from *Klebsiella pneumoniae*. The fourth segment was similar to plasmids pSTA155 (NG041621) and pAPEC-O1-R (DQ517526) and included *aac(6*′*)-Ib-cr* adjacent to an unusual class 1 integron containing *bla*_OXA−1_, *catB3, arr3, qacE*Δ*1*, and *sul1*. Importantly, *oqxAB*, and *aac(6*′*)-Ib-cr* could confer low-level resistance to fluoroquinolones.

### Transcriptional Regulation of ATCC14028 After Acquiring pHXY0908

**Figure 3:**
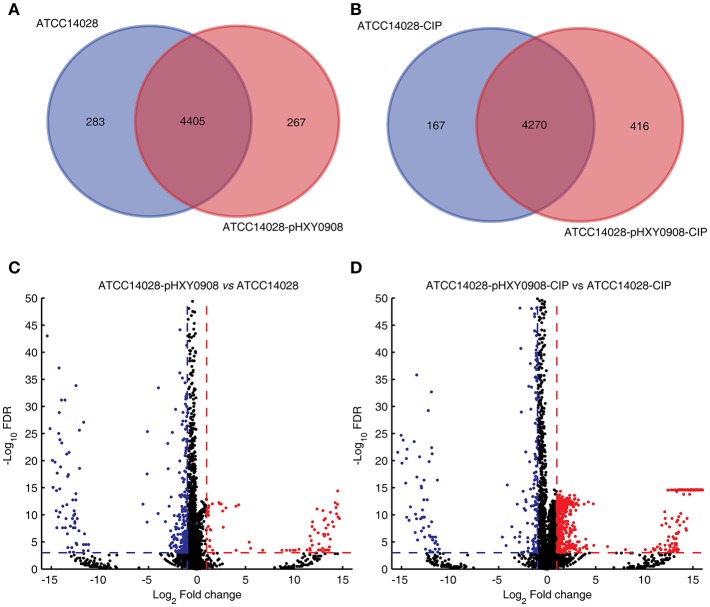
Venn diagram of expressed genes and volcano plot. **(A)** Venn diagram of expressed genes in ATCC14028 and ATCC14028-pHXY0908. **(B)** Venn diagram of expressed genes in ATCC14028-CIP and ATCC14028-pHXY0908-CIP. **(C)** Volcano plot of ATCC14028-pHXY0908 vs. ATCC14028. **(D)** Volcano plot of ATCC14028-pHXY0908-CIP vs. ATCC14028-CIP. The blue plot in volcano plot represented down-regulated genes (Log_2_ Fold change ≤ −1 and *FDR* <0.001). The red plot in volcano plot represented up-regulated genes (Log_2_ Fold change ≥ 1 and *FDR* <0.001).

The transcriptional profiles of ATCC14028 with and without pHXY0908 were obtained using transcriptome sequencing. Both strains were cultured in the presence and absence of 1/2 × MIC concentrations of ciprofloxacin. We quantified the levels of 4,687 and 4,671 genes in ATCC14028 and ATCC14028-pHXY0908, respectively. The 283 genes were only expressed in ATCC14028 and the 267 genes were only expressed in ATCC14028-pHXY0908 (Figure 3A). We found 411 chromosomal DEGs between ATCC14028-pHXY0908 and ATCC14028 (Figure 3C). And 223 plasmid genes were expressed in ATCC14028-pHXY0908. The plasmid situated efflux pump *oqxA* and *oqxB* genes were highly expressed in ATCC14028-pHXY0908. Interestingly, the chromosomal efflux pump gene *acrB* was significantly up-regulated (2.5-fold, *P* <10^−32^) in the plasmid-bearing strain. The *acrA* gene which product composed efflux pump with AcrB protein was also slightly up-regulated (1.6 fold, *P* <10^−32^). The *tolC* gene was also significantly up-regulated as same as *acrB* (2.16 fold, *P* = 1.5 ×10^−11^).

The gene expression profiles of these strains also differed with the strains cultured in the presence of ciprofloxacin. We quantified the expression of 4,473 and 4,686 genes in ATCC14028-CIP and ATCC14028-pHXY0908-CIP, respectively. The 167 genes were only expressed in ATCC14028-CIP and the 416 genes were only expressed in ATCC14028-pHXY0908-CIP (Figure 3B). There were 1029 chromosomal DEGs between ATCC14028-pHXY0908-CIP and ATCC14028-CIP. And 224 plasmid genes were expressed in ATCC14028-pHXY0908-CIP. The *acrB* and *tolC* genes were up-regulated in ATCC14028-pHXY0908-CIP, but we found no difference for *acrA*. Furthermore, we found that many chromosomal efflux pump genes were up-regulated significantly in ATCC14028-pHXY0908-CIP compared with ATCC14028-CIP, for example, *ydgF, ydgE, ybjY, yceE, sugE*, and *yohM* (Figure 3D).

### The GO and KEGG Enrichment Analysis of Chromosomal DEGs of ATCC14028 After Acquiring pHXY0908

**Figure 4:**
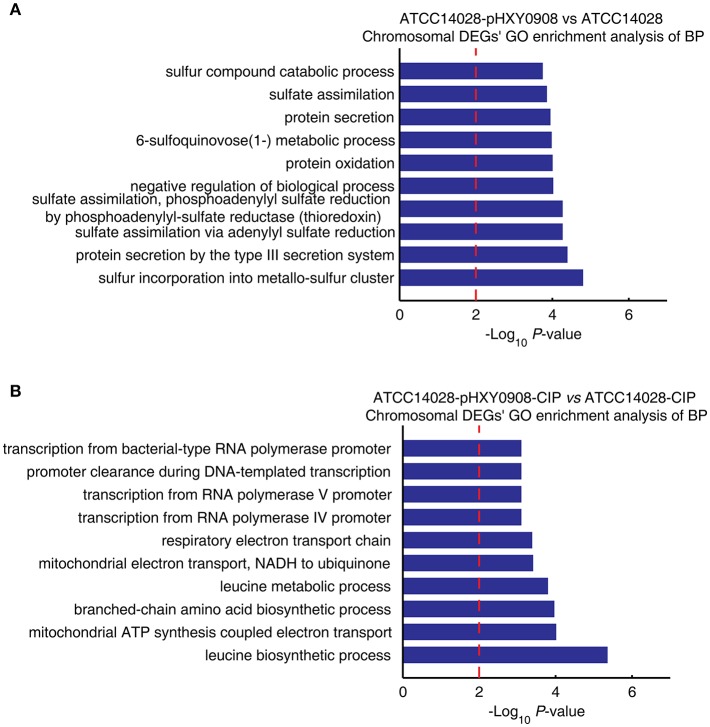
The chromosomal DEGs GO enrichment analysis of biological processes. **(A)** ATCC14028-pHXY0908 vs. ATCC14028 and **(B)** ATCC14028-pHXY0908-CIP vs. ATCC14028-CIP **(B)**. The top 10 biological processes are shown. The dotted red line indicates the *P*-value threshold of 0.01.

The transcriptional profile of ATCC14028 was changed after acquiring pHXY0908. GO enrichment analysis indicated that chromosomal DEGs of ATCC14028-pHXY0908 vs. ATCC14028 were enriched for some biological processes. These included sulfur incorporation into metallo-sulfur clusters, sulfate assimilation, 6-sulfoquinovose (1-) metabolism, sulfur compounds in catabolic process, type III protein secretion (Figure 4A). Interestingly, six biological processes in the top ten enriched biological processes were related to sulfide. pHXY0908 contained the thiol:disulfide interchange protein DsbC that is responsible for the formation of disulfide bonds and this process is related to sulfur metabolism (Mangold et al., 2011). This suggests that ATCC14028 may regulate genes expression to support the plasmid function. In addition, sulfur-mediated biological process could provide a new way for ciprofloxacin biodegradation (Jia et al., 2018).

The chromosomal DEGs of ATCC14028-pHXY0908-CIP vs. ATCC14028-CIP were enriched in four transcriptional biological processes and two electron transport chain processes (Figure 4B). After acquiring pHXY0908, ATCC14028 could activate transcription of resistance genes including efflux pump genes under ciprofloxacin selective pressure. This indicates that pHXY0908 can influence the gene expression of ATCC14028 to resist ciprofloxacin selective pressure.

**Figure 5:**
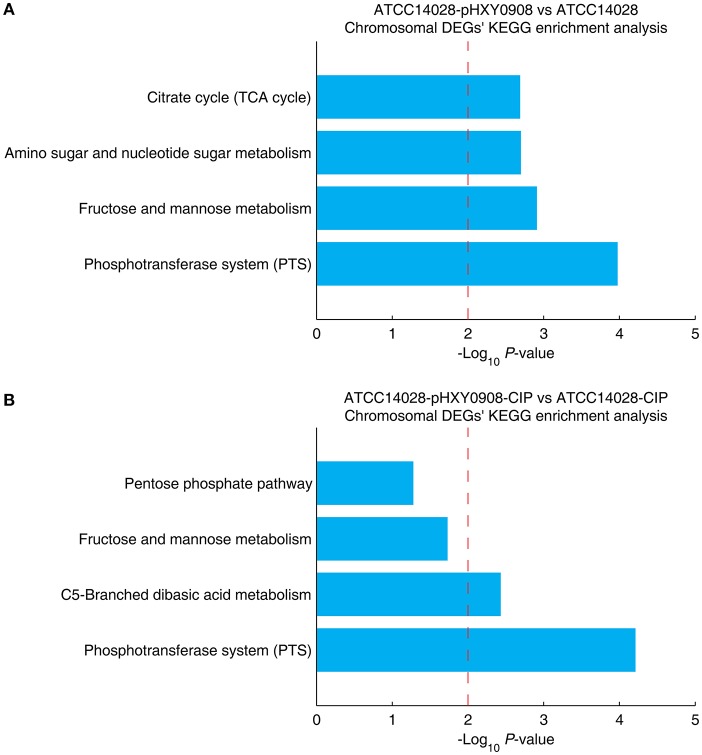
The chromosomal DEGs KEGG pathway enrichment analysis. **(A)** ATCC14028-pHXY0908 vs. ATCC14028 and **(B)** ATCC14028-pHXY0908-CIP vs. ATCC14028-CIP **(B)**. The dotted red line indicates the *P*-value threshold of 0.01.

The chromosomal DEGs of ATCC14028-pHXY0908 vs. ATCC14028 were enriched in four KEGG pathways, including phosphotransferase system (PTS), fructose and mannose metabolism, amino sugar and nucleotide sugar metabolism and citrate cycle (TCA cycle) (Figure 5A). Additionally, the KEGG pathways enrichment analysis for DEGs of ATCC14028-pHXY0908-CIP vs. ATCC14028-CIP is C5-branched dibasic acid metabolism and phosphotransferase system (PTS) (Figure 5B). The phosphotransferase system (PTS) serves as a complex protein kinase system that regulates a wide variety of transport, metabolic and mutagenic processes as well as the expression of numerous genes (Saier, 2015). That indicated the pHXY0908 influenced the gene expression, the substance metabolism and energy metabolism in the ATCC14028.

### The Functional Protein Association Networks Analysis for DEGs of ATCC14028 After Acquiring pHXY0908

**Figure 6:**
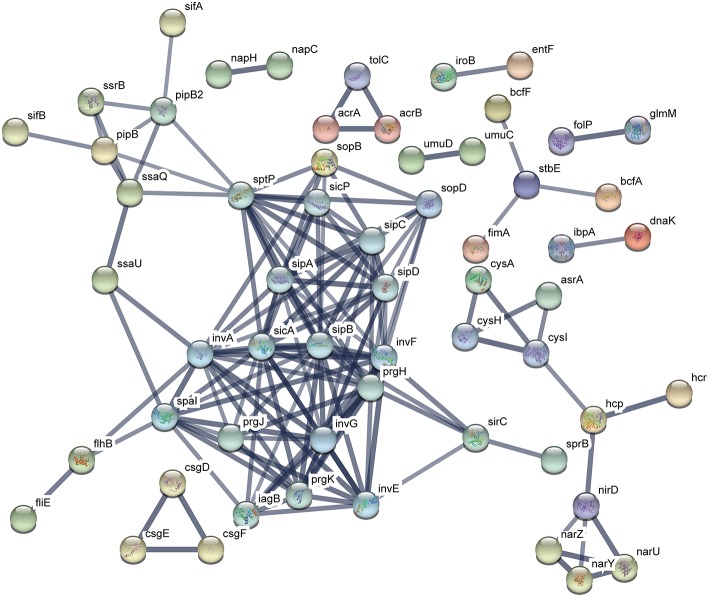
Functional protein association networks of chromosomal DEGs after acquiring pHXY0908. Each node is a protein. Each edge represents protein-protein associations. The thicker edges indicate higher levels of confidence.

The functional protein association networks were constructed by chromosomal DEGs of ATCC14028 after acquiring pHXY0908. A majority of DEGs were constructed into two main networks. This suggests pHXY0908 may systematically regulate ATCC14028 functional protein association networks rather than at an independent gene expression level. The largest sub-network that included the genes *spal, invA, sicA*, and *sipB* is related to invasion and type III secretion systems. Another sub-network that included *cysA, cysH*, and *cysl* was related to sulfur metabolism. These two main functional networks were consistent with the GO enrichment analysis and pHXY0908 function (Figure 6). Additionally, the networks of ATCC14028-pHXY0908-CIP vs. ATCC14028-CIP DEGs also contained the sub-networks related to invasion and type III secretion systems, sulfur metabolism, energy metabolism, and other substance metabolism (Figure S2).

### RNA-seq Data Validated by qRT-PCR

Five randomly selected DEGs that including *dam, csgD, csgE, STM14-1453*, and *acrB*, and five genes which we interested, including *acrA, tolC, yceE, sicA*, and *cysA*, were validated by using qRT-PCR. The qRT-PCR results had a good correlation with different expression analysis (Figure S3, *R* = 0.9793, *P* = 7.7606 ×10^−7^ in ATCC14028-pHXY0908 vs. ATCC14028; *R* = 0.9761, *P* = 1.3851 ×10^−6^ in ATCC14028-pHXY0908-CIP vs. ATCC14028-CIP). This indicated that the RNA-seq and the associated data analysis represented actual gene expression changes.

## Discussion

This study demonstrated that an MDR IncHI2 type plasmid encoding OqxAB could contribute to the survival under ciprofloxacin selective pressure of *S*. Typhimurium strains that had acquired plasmid pHXY0908. Although the MIC and MPC increased after acquiring pHXY0908, the same MPC/MIC ratios for both ATCC14028 and ATCC14028-pHXY0908 indicated that pHXY0908 did not increase ability of ciprofloxacin for selecting resistant mutation in ATCC14028. The time-kill curve assays demonstrated that ciprofloxacin concentrations >MIC values for *S*. Typhimurium ATCC14028 were lethal while the plasmid-bearing strain could survive up to 4 × MIC. Thus, we further explored the implied survival mechanism of *S*. Typhimurium under drug pressure after acquiring an *oqxAB*-positive IncHI2 type plasmid. The same MPC/MIC suggested that the genes expression regulation may be the potential mechanism instead of genes mutation.

Firstly, we determined the complete nucleotide sequence of plasmid pHXY0908.

DNA sequencing further confirmed that this plasmid belonged to the IncHI2 group and showed high similarity to the backbone region of R478, one of the prototypes of IncHI2 plasmid groups (Garcia-Fernandez and Carattoli, 2010). In addition to the conserved backbone, pHXY0908 harbored a multidrug resistance region composed of *oqxAB, aac(6*′*)-Ib-cr* as well as nine other ARGs and included a wide range of mobile genetic elements and notably, IS*26*. Except for *oqxAB* and *aac(6*′*)-Ib-cr*, none of the other known genes are involved in fluoroquinolone resistance although many hypothetical proteins were found on pHXY0908. In addition, pHXY0908 showed a surprisingly high degree of homology (100% coverage, 99% identity) with the IncHI2 plasmid pHK0653 from an *S*. Typhimurium strain of human origin and pHK0653-like plasmids that were identified as the key vectors responsible for *oqxAB* transmission among *Salmonella* species (Wong et al., 2016).

Plasmid pHXY0908 harbored diverse ARGs including *oqxAB* and *aac(6*′*)-Ib-cr* that could confer only low-level fluoroquinolone resistance. However, the gene features of pHXY0908 cannot explain the reason for ATCC14028 survival under the lethal concentrations of ciprofloxacin up to 4 × MIC. To study the influence of pHXY0908, we measured the transcriptional profiles of ATCC14028 with and without pHXY0908 cultured in the presence and absence of 1/2 MIC concentrations of ciprofloxacin. ATCC14028 could not grow at >1 × MIC, so we selected a 1/2 MIC level of ciprofloxacin to study the transcriptional regulation of ATCC14028 by pHXY0908. The 1/2 MIC concentrations allowed bacterial growth but also induced stress responses that are sub-MIC and often used in the study of antibiotic resistance (Patkari and Mehra, 2013; Heo et al., 2014; Zhong et al., 2015; Aedo and Tomasz, 2016). The transcriptome data show that 283 chromosomal genes were not expressed after ATCC14028 acquiring pHXY0908. Similarly, there are 167 chromosomal genes were not expressed in ATCC14028-pHXY0908-CIP compared with ATCC14028-CIP. The bacteria will synthesize proteins encoded by plasmid leading the cost of resources. The bacteria may turn off some chromosomal genes expression to fit this situation. These genes were included in DEGs to analyze the function. Due to the ciprofloxacin inhibits the activity of gyrase and topoisomerase IV, we analyzed the expression of *gyrA, gyrB, parC*, and *parE*. These four genes were not differently expressed in ATCC14028-pHXY0908 compared with ATCC14028. The *gyrA* was down-regulated in ATCC14028-pHXY0908-CIP compared with ATCC14028-CIP. However, other three genes were not differently expressed in ATCC14028-pHXY0908-CIP compared with ATCC14028-CIP. The gyrA down-regulated in ATCC14028-pHXY0908-CIP compared with ATCC14028-CIP may due to the resistant proteins encoded by pHXY0908 could decreased the ciprofloxacin pressure in the cell. The GO enrichment analysis of DEGs indicated that pHXY0908 influenced chromosomal gene expression to support plasmid function and resist ciprofloxacin selective pressure. This indicated an interaction between the chromosome and plasmid. And the KEGG pathway enrichment analysis of DEGs indicated that pHXY0908 influence ATCC14028 genes expression and metabolism. Furthermore, the pHXY0908 systematically regulated ATCC14028 functional protein association networks. Intriguingly, the chromosomal efflux pump genes were up-regulated after acquiring the plasmid and these included *acrB, acrA*, and *tolC*. Except for AcrAB-TolC efflux pump, the *ydgF, ydgE, ybjY, yceE, sugE*, and *yohM* were especially up-regulated when cultured under the 1/2 MIC levels of ciprofloxacin. The multidrug efflux pump AcrAB-tolC system decreases susceptibility to fluoroquinolones (Piddock, 1999) and *yceE* encoding the multidrug transporter subunit MdtG contributes to fluoroquinolone-resistance (Fàbrega et al., 2010). The *ydgF* and *ydgE* encoded MdtI and MdtJ, respectively. These two proteins compose multidrug efflux pump MdtIJ which excretes polyamine (Higashi et al., 2008; Leuzzi et al., 2015). The *ybjY* codes macrolide transporter subunit MacA. The *sugE* was reported the contribution for tributyltin (TBT) and antimicrobial resistance (He et al., 2011; Cruz et al., 2013). And the *yohM* encodes a membrane-bound polypeptide conferring increased nickel and cobalt resistance (Rodrigue et al., 2005). The up-regulation of chromosomal efflux pump genes may be caused by the accumulation of intermediate metabolites for plasmid DNA replication and proteins synthesis. In addition, another potential mechanism is some plasmid proteins regulated chromosomal genes. It is worthy of further investigations. The chromosomal efflux pump up-regulated by pHXY0908 may be the one of the reasons for survival under the lethal concentrations of ciprofloxacin. Excepted the efflux function, the synthesis of chromosomal efflux pump proteins, the activity of efflux pumps and the encoding of plasmid genes may cost much energy, so that the growth of ATCC14028 may be influenced. The slow grown speed lead to the tolerance to fluoroquinolone (Evans et al., 1991; Brauner et al., 2016). The change of ATP level and grown rate of the ATCC14028 after acquiring pHXY0908 may be measured in the further researches. However, the specific molecular mechanism of the tolerance phenotype is not clear. In the further investigations, we will use functional genome technologies combined our plasmid gene expression profiles to detected the plasmid gene caused the tolerance to ciprofloxacin.

Taken together, we found that chromosomal genes were systematically regulated after ATCC14028 acquired the IncHI2 type MDR plasmid pHXY0908. ATCC14028 could survive under the ciprofloxacin lethal concentrations may be attributed to an up-regulation of chromosomal efflux pump genes after acquiring pHXY0908.

## Data Availability

The raw data supporting the conclusions of this manuscript will be made available by the authors, without undue reservation, to any qualified researcher.

## Author Contributions

XLL carried out the study design, data analysis, and manuscript writing. XW carried out the sequence experiments and was involved in the preparation of the manuscript. XL and JX carried out the RT-qPCR experiments. LF, JS, XPL, and YL contributed to the study design. All authors approved it for publication.

### Conflict of Interest Statement

The authors declare that the research was conducted in the absence of any commercial or financial relationships that could be construed as a potential conflict of interest.
