# Supplementary material for: oqxAB-Positive IncHI2 Plasmid pHXY0908 Increase Salmonella enterica Serotype Typhimurium Strains Tolerance to Ciprofloxacin
Source: Front Cell Infect Microbiol. 2019 Jul 3;9:242. doi: 10.3389/fcimb.2019.00242 (PMC6617520; doi:10.3389/fcimb.2019.00242)
Supplement: Supplementary file 1 [file Data_Sheet_1.docx]

**Supporting Information for**

***oqxAB*-positive IncHI2 Plasmid pHXY0908 Increase** ***Salmonella enterica* Serotype Typhimurium Strains Tolerance to Ciprofloxacin**

**Number of tables: 1**

**Number of figures: 3Table S1.** RT-qPCR primers used in this study.

| Gene | Forward Primer (5’-3’) | Reverse Primer (5’-3’) |
| --- | --- | --- |
| *dam* | GGGCGACCGGAGAATAGAAG | ACACAGACTCAGCGCTTACC |
| *csgD* | CCACGTGTTCCTGGTCTTCA | CGGCCGGTTGCATTGTTTTA |
| *csgE* | AGAGACGGTCTGGTCGGTTA | CTGTTCGCTACCGGAAACCT |
| *STM14-1453* | GCAAATCCGTTGGTGGTGAC | CGGCGTTACCGTGATAGTGA |
| *acrB* | AAGAGCACGCATCACTACAC | CGCTTCGGACATCACGTAAA |
| *acrA* | GAAGATGGCGCGCAAAGTAA | AATGACTTCCTGCGCCTGAA |
| *tolC* | CGTAACCTGTCGCTGTTGCA | CTGCCCCATGTTGCTATCGT |
| *yceE* | TTGGCGTTGGGAATAAATCC | GGTCCGGGCTGGTCTTTAGT |
| *sicA* | CCATGGTGTAATCGGGATTGT | TTTGGGATGCCGTTAGTGAAG |
| *cysA* | GCAATTGCGTCACTTTCGTTT | TCCACGGTACGGACGTTAGC |
| 16S rRNA | ATTAGATACCCTGGTAGTCCACGC | TTGCGGGACTTAACCCAAC |


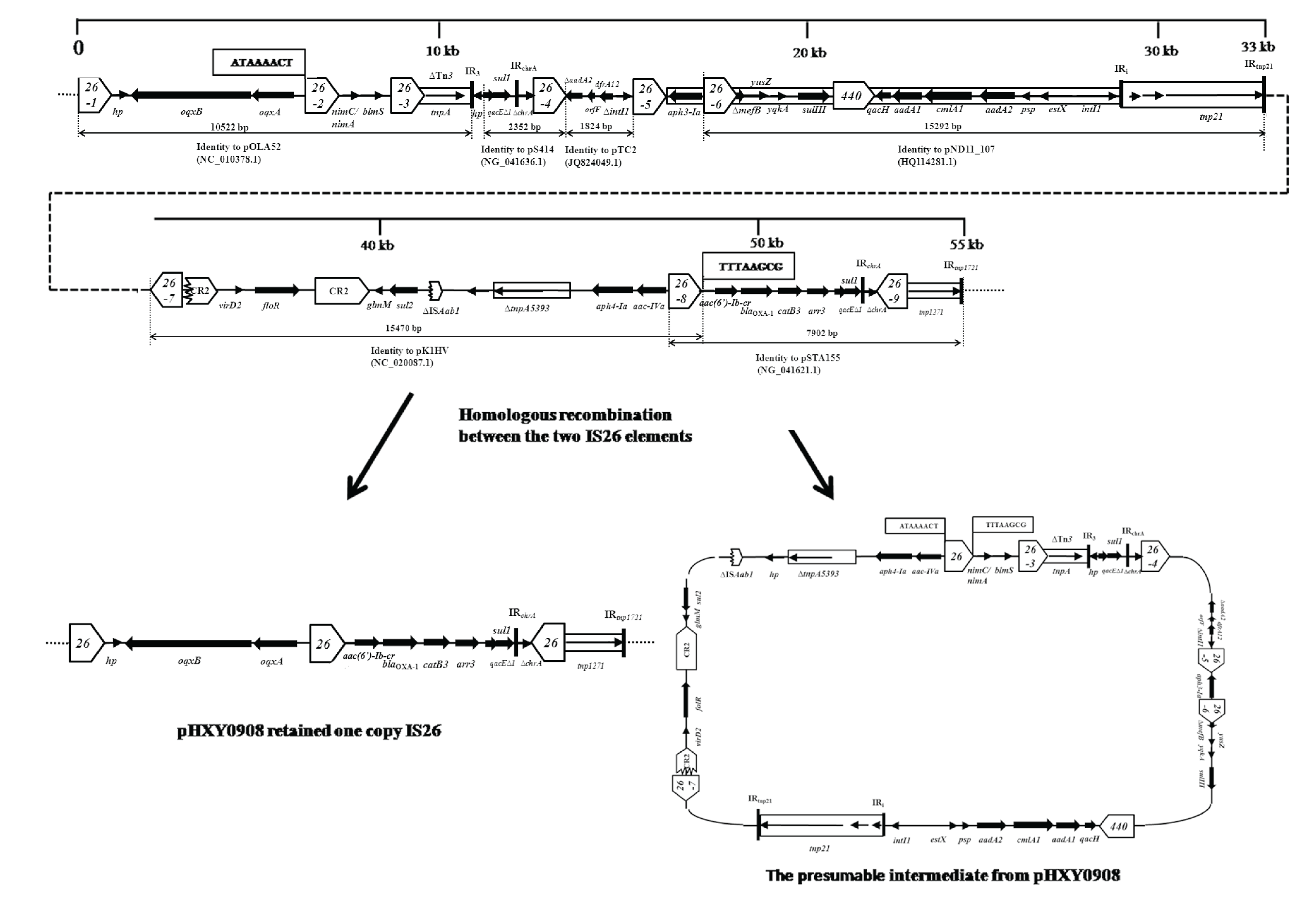


Figure S1. Schematic presentation of the multi-drug resistance intermediate formation in pHXY0908. Arrows indicate ORFs and genes, and their transcriptional orientation. Antibiotic resistance genes are shown as thick black arrows labeled with the gene name. The dotted line indicates plasmid backbone. IS are shown as boxes labeled with the name/number of the IS. IS flanked by direct repeats (DRs) have generally been omitted. Tall bars represent Tn*3*-like 38-bp IR or IRi of class 1 In/Tn, as indicated.


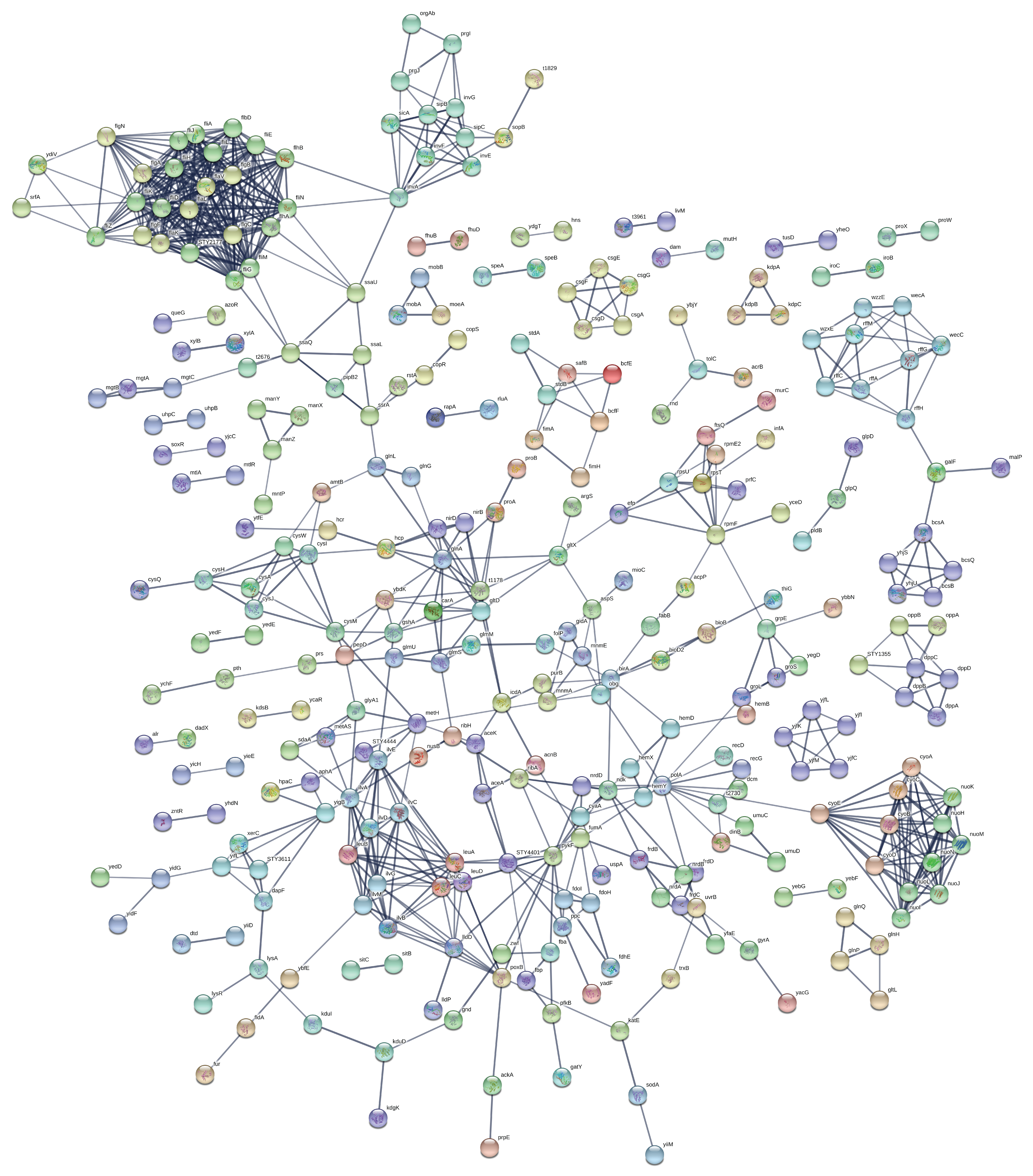


**Figure S2.** Functional protein association networks of chromosomal DEGs of ATCC14028-pHXY0908-CIP vs ATCC14028-CIP. Each node is a protein. Each edge represents protein-protein associations. The thicker edges indicate higher levels of confidence.


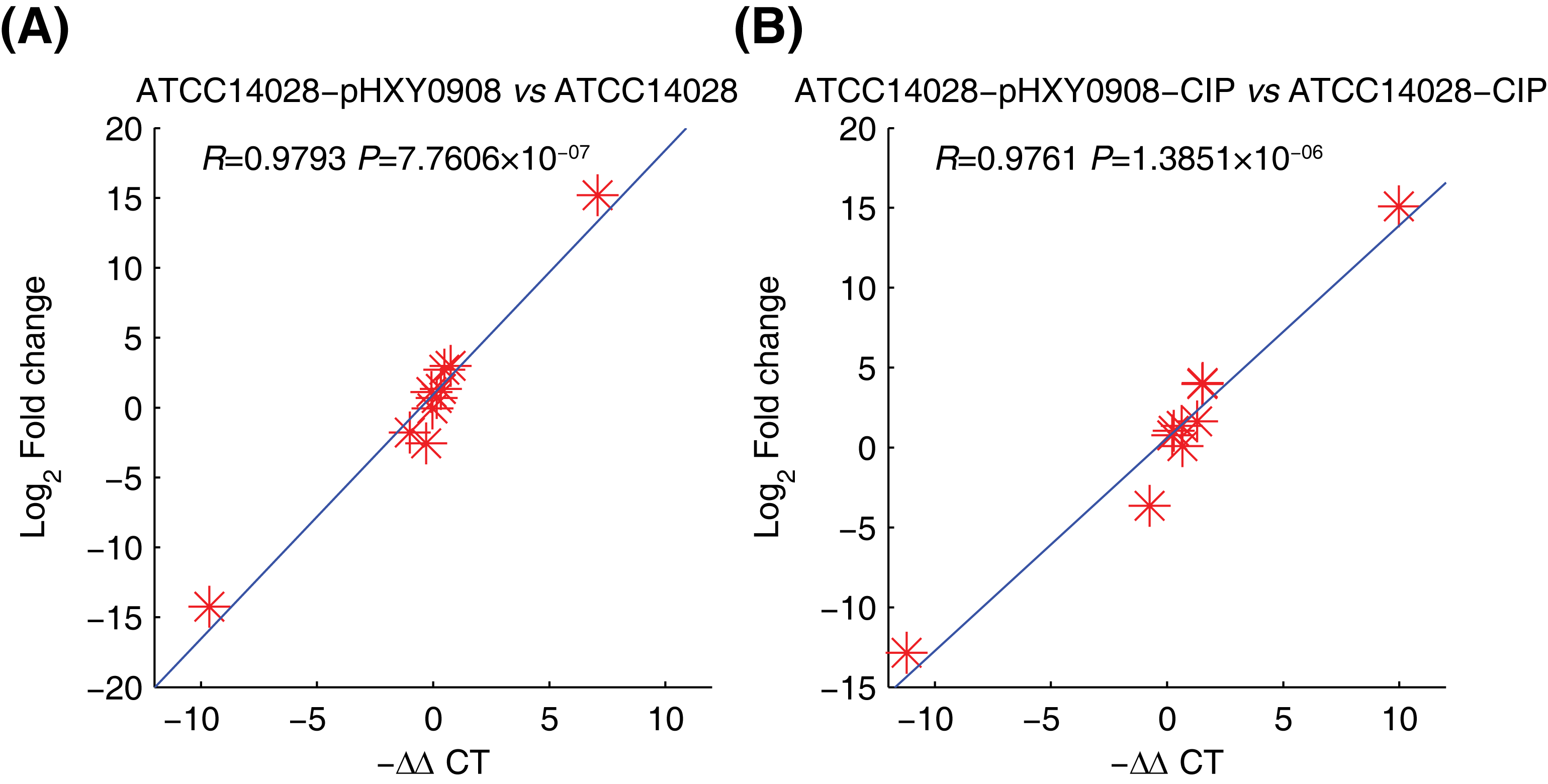


**Figure S3.** Validation of DEGs by RT-qPCR. The X-axis represents the RT-qPCR results (-ΔΔCT) and the Y-axis represents different expression analysis result (Log_2_ Fold change). The correlations between RT-qPCR and DEG analysis in both ATCC14028-pHXY0908 *vs* ATCC14028 (A) and ATCC14028-pHXY0908-CIP *vs* ATCC14028-CIP (B) were good. That means the DEGs analysis could show the real change of gene expression.
